# Brain-Derived Neurotrophic Factor and Diabetes

**DOI:** 10.3390/ijms21030841

**Published:** 2020-01-28

**Authors:** Olga Rozanska, Aleksandra Uruska, Dorota Zozulinska-Ziolkiewicz

**Affiliations:** Department of Internal Medicine and Diabetology, Poznan University of Medical Sciences, ul. Mickiewicza 2, 60-834 Poznań, Poland; olgalehman@wp.pl (O.R.); zozula@box43.pl (D.Z.-Z.)

**Keywords:** brain-derived neurotrophic factor (BDNF), neurotrophins, diabetes mellitus, physical activity, neuroprotection, insulin resistance

## Abstract

Diabetes and its chronic complications still represent a great clinical problem, despite improvements made in the diagnosis and treatment of the disease. People with diabetes have a much higher risk of impaired brain function and psychiatric disorders. Neurotrophins are factors that protect neuronal tissue and improve the function of the central nervous system, and among them is brain-derived neurotrophic factor (BDNF). The level and function of BDNF in diabetes seems to be disturbed by and connected with the presence of insulin resistance. On the other hand, there is evidence for the highly beneficial impact of physical activity on brain function and BDNF level. However, it is not clear if this protective phenomenon works in the presence of diabetes. In this review, we summarize the current available research on this topic and find that the results of published studies are ambiguous.

## 1. Diabetes Mellitus and Brain Function

Every year, there is a growing incidence of both type 1 and type 2 diabetes. Despite different causes of the disease (failure in insulin production or insulin resistance), the effect of the disease remains the same: hyperglycaemia [1]. It has been observed that people with diabetes caused by autoimmune reactions can also have or develop insulin resistance, typically linked to type 2 diabetes. Decreased insulin sensitivity impairs management of the disease and increases the risk of chronic complications. As a consequence, it worsens the prognosis of people with diabetes. Despite increasingly better tools for the control and regulation of the glucose level in blood, the percentage of good metabolic management of the disease is poor. Within years, diabetes may lead to decreased quality of life due to the development of numerous complications, such as eye, kidney, nerve and heart damage [1]. Finally, it involves premature death.

Diabetes also significantly influences the function of the central nervous system (CNS). It is a well-known fact that diabetes increases the risk of neurologic and psychiatric diseases. In Latinos over 60 years old, diabetes causes a higher risk of depression symptoms [2]. It was also proved that women in Australia who had diabetes experienced anxiety disorders more often than healthy women [3]. Moreover, children with type 1 diabetes were found to very often also suffer from psychiatric diseases, which in their case correlated with higher concentrations of HbA1c (glycated hemoglobin) [4]. Type 2 diabetes is also related to increased risk of Alzheimer’s disease and vascular dementia [5]. Higher glucose concentration in brain tissues and higher insulin resistance correlated with the severity of Alzheimer’s disease and the expression of its symptoms [6]. Moreover, there is a hypothesis that Alzheimer’s disease is a diabetes type 3. Insulin resistance is related to the production of beta-amyloid deposits in the CNS [7]. It was also proven that in patients with Parkinson’s disease, insulin resistance occurred more often in a group of patients with dementia than in patients suffering from Parkinson’s disease but without coexisting dementia [5]. There are also data about the role of neurotrophins and metabolic effects in the course of anorexia nervosa [8], which occurs more often in the diabetic population.

Factors that could contribute to improving the prognosis and metabolic control of diabetic patients are still being searched for. It is known, however, that one such factor is systematic physical activity. The exact mechanisms of its influence on glycemia remain unexplored. There are numerous premises that increased secretion of neurotrophins might be one such mechanism. Apart from this, physical activity has a positive impact on CNS function, as it improves cognitive functions, improves memory, and decreases the risk of dementia.

The purpose of this review article is to explore the connection between brain-derived neurotrophic factor (BDNF) and diabetes. In particular, we explore the hypothesis that increased levels of neurotrophins in target tissues of people with diabetes, induced by physical activity, will improve the course of the disease.

## 2. Neurotrophins

Neurotrophins are a family of proteins that consist of nerve growth factor (NGF), brain-derived neurotrophic factor (BDNF), and neurotrophins NT-3 and NT-4/5. Neurotrophins are responsible for increasing the survival of neurons and their damage resistance [9].

BDNF via its receptors, p75 NT receptor (p75NTR) and tyrosine kinase receptor B (TrkB), affects neurons in multiple ways. Mature BDNF acts on neurons mainly via the Trk receptor [10]. BDNF can stimulate nerve regeneration and is responsible for the development, plasticity and support of the nervous system [9,10]. 

Neurotrophins are produced mainly by the CNS, but also by peripheral tissues [11]. BDNF and also its mRNA as well as TrkB are largely found in the hypothalamus, limbic system and other areas of the brain, such as hippocampus nuclei [10,12]. What is more, BDNF is also expressed in the skeletal muscle, endothelial cells, liver, adipose tissues, and activated immune cells [10,13]. Blood serum and plasma also contain BDNF. Studies have shown that peripheral BDNF is stored in platelets [10].

**Figure 1:**
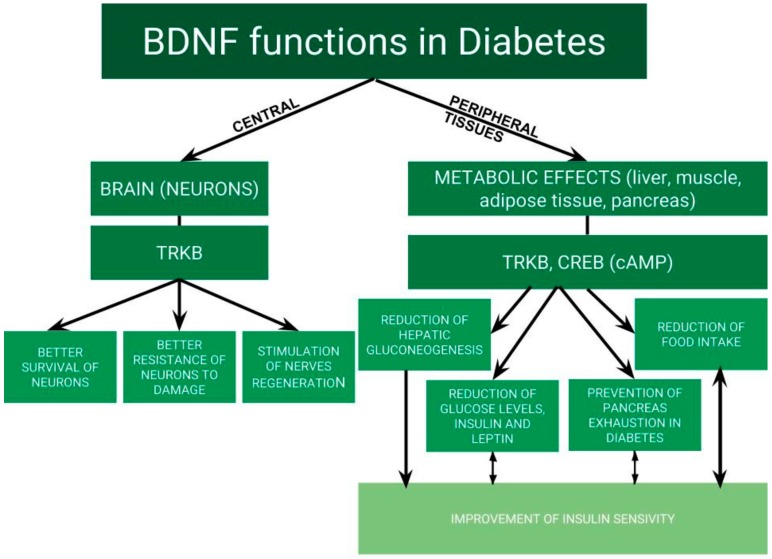
Functions of brain-derived neurotrophic factor (BDNF) in diabetes.

In addition to its central effects on brain tissue, BDNF has a role in energy homeostasis in humans [9]. Thus, the amount of data about the role of BDNF in metabolic control, especially glucose metabolism and insulin resistance, is still increasing [14,15]. BDNF is also named “metabokine” because of its effects on glycemia, lipid profile and energy homeostasis. BDNF levels are impaired in atherosclerosis, acute coronary syndrome and metabolic syndrome [16,17]. Moreover, altered BDNF level is related to increased appetite, obesity, type 2 diabetes, depression, as well as Alzheimer’s and Parkinson’s diseases [17,18,19]. BDNF has become an interesting target as a treatment option. The functions of BDNF in diabetes are presented in Figure 1.

The factors influencing BDNF concentration in target tissues are still being researched. Komori et al. investigated the effect of intravenous leptin injection in mice. Their results suggest that leptin induces BDNF expression in the dorsomedial part of the ventromedial hypothalamic nucleus [13]. There are ongoing studies on the relationship between BDNF and adipocytokines, such as leptin, resistin, and adiponectin. 

## 3. Neurotrophins and Insulin Resistance

Insulin resistance is a factor that has a significant influence on metabolic control and the development of diabetes and its chronic complications. The relationship between neurotrophins and insulin resistance (IR) has long been studied. The results of published studies are inconclusive.

### 3.1. Experimental and Animal Studies

In experimental and animal studies, it was shown that an infusion of BDNF in obese diabetic mice reduced the glucose level in blood. This phenomenon did not occur in non-diabetic mice. The mechanism of this effect is probably an improvement in insulin functioning in peripheral tissues caused by BDNF [20]. Smieszek et al. determined the serum level of BDNF in two groups: mice receiving metformin for eight weeks at a dose of 2.8 mg/day, and a control group (receiving a placebo). The administration of metformin (a medicine that decreases insulin resistance) stimulated an increased release of BDNF into the serum [21]. Duan et al. described a way to increase the level of neurotrophins. In an experiment on mice, they introduced dietary restrictions that stimulated BDNF production in brain cells. These restrictions decreased the concentrations of glucose, insulin, and leptin in mice whose level of these factors was increased. This effect might have been significantly influenced by BDNF [22]. Mitsugu et al. carried out research on obese diabetic mice (for four weeks, BDNF or a placebo was subcutaneously administered to each of two groups). The experiment showed that the BDNF group had a lower glucose concentration than the placebo group. As far as insulin level in plasma is concerned, there was no significant difference between the two groups after the experiment was finished. BDNF significantly lowered the concentration of pancreatic glucagon and increased the concentration of pancreatic insulin. Histologic analysis showed that BDNF administration had no impact on increasing the overall number of pancreatic islets. It was showed that the BDNF group had more beta cells producing insulin in pancreatic islets than the placebo group. The BDNF group also had a lesser area of non-producing insulin cells than the placebo group. This study suggests that BDNF administration may prevent pancreas exhaustion in diabetic mice [23]. Insulin resistance involves not only incorrect glucose utilization in peripheral tissues, but also increased hepatic glucose production. Studies showed that the administration of exogenous BDNF in obese rats generates ejection of hepatic glucokinase, as well as improving glucokinase activity, which reduces hepatic gluconeogenesis and improves insulin sensitivity. An increase in hepatic glucokinase activity may decrease the levels of fasting glucose and postprandial blood glucose and decrease hyperinsulinemia [24]. In a study with streptozotocin mice, animals received daily intracerebroventricularor or into the ventromedial hypothalamic nucleus injections of either BDNF or its vehicle. The authors revealed that BDNF decreased hyperglycemia, independent of changes in food intake, and lowered blood glucose levels in an insulin-independent manner. BDNF suppressed hepatic glucose production through the inhibition of glucagone [25]. 

In summary, decreased insulin resistance stimulates the increased release of BDNF into the serum [21]. In animal studies, dietary restrictions resulted in reduced glycemia through the stimulation of the production of BDNF in brain cells [22]. Exogenous infusions of BDNF reduced blood glucose levels [20,23,24,25]. BDNF has a beneficial impact on metabolic control in animals.

### 3.2. Type 2 Diabetes Mellitus

In patients with type 2 diabetes, the concentration of BDNF is lower than in healthy control groups. Moreover, it was shown to be lower in diabetic men than in diabetic women. This difference was not observed in the control group. The concentration of BDNF correlates positively with immunoreactive insulin and HOMA-IR (homeostatic model assessment for insulin resistance) in women with type 2 diabetes. This effect was not observed in men with type 2 diabetes. Differences between the sexes in the results of the above-mentioned studies may be caused by concentrations of estrogens and other sex hormones that influence glucose metabolism and contribute to the development of insulin resistance. Moreover, the authors observed that in women with type 2 diabetes, the longer the duration of diabetes, the lower the BDNF concentration [26]. The same observations were seen in a Chinese population: BDNF serum levels in patients with type 2 diabetes mellitus were significantly lower than in a healthy control group [27]. 

Boyuk et al. had another opinion on BDNF levels in patients with diabetes. They proved that the serum of patients with type 2 diabetes contained much higher levels of BDNF than the serum of the control group. The control group had significantly lower Body Mass Index (BMI), waist circumference, blood pressure, fasting glucose and insulin concentrations, HOMA-IR, concentration of HbA1c, and C-reactive protein (CRP) than patients with type 2 diabetes. In patients with type 2 diabetes, BDNF concentration in serum correlated positively with HOMA-IR (as in the previous study), as well as with triglyceride level and white blood cells (WBC) level [28]. Suwa et al. have a similar opinion to that of Boyuk et al. They studied the relationship of BDNF concentration in Japanese women newly diagnosed with type 2 diabetes and a control group consisting of women with normal glucose tolerance. BDNF concentration was much higher in patients with type 2 diabetes than in the healthy control group. In patients with diabetes, the authors also showed correlations between BDNF concentration and BMI, percentage of body fat, subcutaneous fat area based on CT (computed tomography) scan, triglyceride level in serum, fasting glucose level, and HOMA-IR. A negative correlation between BDNF and age was also observed [17]. On the other hand, Lee et al. proved that a control group and adolescents with type 2 diabetes mellitus had similar resting BDNF levels [29].

To conclude, there are different opinions about serum BDNF levels in patients with type 2 diabetes. It has been proven that the concentration of BDNF in the serum depends on the duration of the disease. There is a hypothesis that in obese patients, the BDNF level increases to improve their metabolism and decrease their food intake [17]. It was proven that BDNF infusion reduces food intake and body mass [30]. This phenomenon could have a positive effect on the course of the disease in obese patients with type 2 diabetes.

### 3.3. Type 1 Diabetes Mellitus

There are only few data about BDNF and type 1 diabetes, mainly experimental data. Tonoli et al. revealed that serum BDNF levels were significantly higher in people with type 1 diabetes compared with the healthy control group. In both groups, BDNF increased after exercise. Thus, despite impaired resting levels of BDNF, promising conclusions were found that physical activity had an impact on BDNF, increasing it to levels comparable to those found in healthy subjects [31]. Reports on the relationship between IR and neurotrophins in patients with type 1 diabetes showed significantly lowered BDNF concentration in serum in the group with insulin resistance, assessed during hyperinsulinaemic–euglycaemic clamp. There was a strong, positive correlation between BDNF and glucose disposal rate and a significant relationship between clamp results and BDNF, adjusted for sex, duration of diabetes and mean HbA1c [32].

Finally, a very promising hypothesis about the role of BDNF in the regeneration of beta cells has arisen. Neurotrophins may act through neurogenin (Ngn)-3 gene expression, stimulate the regeneration of damaged pancreatic β-cells and restore insulin secretion [33]. 

In summary, there is a need for further studies about the role of BDNF level in serum among patients with type 1 diabetes. It has been proven that the longer the duration of metabolic disorders and the higher the IR intensity, the lower the concentration of neurotrophins. Various questions arise then, including which of these effects is first—does IR disturb neurotrophin secretion or does low neurotrophin level intensify the IR effect? Furthermore, how can it be used in the clinical management of diabetes?

It was proved that physical exercise increases the secretion of BDNF into the serum in people with type 1 diabetes [31]. Therefore, recommending physical exercise seems to alleviate the course of the disease, not only according to the metabolic control and decreased risk of chronic complications but also supporting the neuroregeneration and protection of the CNS in this group.

The relationship between BDNF and decreased insulin sensitivity is probably mediated through adipocytokines.

### 3.4. BDNF and Adipocytokines

Numerous studies are ongoing regarding the association of adipocytokines with BDNF. An et al. presented research on the relationship between the expression of BDNF and the leptin effect regulating mood in the pathophysiology of major depressive disorder. The authors recruited patients with major depressive disorder and healthy controls. Patients suffering from major depressive disorder were treated with standard pharmacotherapy for 12 weeks. In the results of their research, the ratio of pre-treatment pBDNF to leptin was significantly lower in patients with major depressive disorder compared to healthy controls. Moreover, a higher ratio of pre-treatment pBDNF to leptin was associated with a greater treatment response in major depressive disorder [34]. Eyileten et al. investigated, among others, the relationship between serum BDNF levels and serum adipocytokine levels. In their research, they proved that adiponectin levels were significantly associated with serum BDNF levels. In their multivariate backward stepwise analysis, they showed that CADP-CT (collagen-adenosine diphosphate closure time) >141 s; adiponectin concentration >4.22 µg/mL; total cholesterol and low-density lipoprotein levels were independently associated with serum BDNF levels above the median [35].

## 4. BDNF and Chronic Complications of Diabetes 

Despite huge progress in the diagnosis and treatment of diabetes, its chronic complications still remain an important clinical problem. Diabetes is a leading cause of blindness and non-traumatic foot amputations. It is also responsible for kidney failure and the need for renal replacement therapy as the most common reason. Finally, diabetes is an independent risk factor of cardiovascular diseases, which are the main cause of death in this group of patients. 

The traditional risk factors for diabetic angiopathy are hyperglycemia, duration of the disease, dyslipidemia, smoking, and insulin resistance. New, non-traditional risk factors and markers are still sought after. Among those new factors, neurotrophins have a place. 

In the research described below, the authors mainly focused on the serum level of BDNF as a risk factor for diabetes complications. The authors also noticed the potential therapeutic role of BDNF.

Ola et al. in their research investigated the serum level of BDNF in blood samples from 88 individuals (47 patients suffering from proliterative diabetic retinopathy, 22 diabetic patients with no retinopathy, 19 healthy participants). Patients suffering from proliferative diabetic retinopathy had a decreased level of BDNF in the serum compared to a healthy control group and diabetic patients with no retinopathy.

Ola et al. also conducted tests on rats. They used adult male Sprague–Dawley rats, 9–10 weeks of age. Streptozocin was injected intraperitoneally and citrate buffer was administered to non-diabetic animals in order to induce the development of diabetes. After 3/10 weeks, the rats were killed and their retinas were examined. Then, their blood was collected from the heart (control/diabetic animals). A significant difference in the serum level of BDNF was evident in the 10-week group with diabetes (there was no significant difference in the three-week group). The serum level of BDNF was higher in the control group than in the 10-week diabetes rat group. Moreover, the level of BDNF in the retinal homogenate of both the three-week and 10-week diabetes rat groups was lower than in the control group [36].

Kaviarasan et al. were searching for risk factors of diabetic retinopathy. They conducted a study on a group of 114 participants. The majority of participants were people suffering from type 2 diabetes (27 individuals had no complications, 30 patients had non-proliferative diabetic retinopathy, and 30 patients had proliferative diabetic retinopathy), but they also examined 27 healthy participants as a control group. They revealed that BDNF levels were lower in patients suffering from proliferative diabetic retinopathy and non-proliferative diabetic retinopathy compared to the control group. What is more, this research proved that BDNF has a positive correlation with interleukin-10. This study shows that a low serum level of BDNF is a risk factor for diabetic retinopathy [37].

Seki et al. investigated the effects of intraocular administration of BDNF to streptozotocin-induced diabetic rats. In their research, they used adult male Wistar rats, nine weeks of age. Streptozocin was injected intraperitoneally, and non-diabetic animals were administered with a citrate buffer. Diabetes was confirmed by determining the level of glucose in the blood. The rats also had BDNF injected into their vitreous cavities. Rats were killed four weeks after the streptozicin injection, and their retinas were examined. Seki et al. suggested that dopaminergic amacrine cells degenerate in the retinas of diabetic rats. They proved that non-diabetic rats had a higher tyrosine hydroxylase density (marker for retinal dopaminergic amacrine cells) in their retinas than diabetic animals. It was shown that the protein and mRNA levels of BDNF in the retinas of diabetic animals were lower than those of normal control rats. Injection of BDNF into the vitreus cavities of diabetic rats prevented dopaminergic amacrine cells from degeneration. This study suggests that BDNF may have a role in the treatment of early retinal neuropathy [38].

Sun et al. in their experiment investigated blood samples from 110 healthy individuals, 83 patients suffering from diabetes mellitus type 2, and 65 patients with diabetic peripheral neuropathy. They tried to find correlations between serum BDNF levels and clinical parameters in diabetic peripheral neuropathy. Serum levels of BDNF in the diabetes type 2 group and the control group were higher compared to the diabetic peripheral neuropathy group. Serum levels of BDNF in the control group were higher compared to the patients with diabetes mellitus type 2. Qin Sun et al. revealed that BDNF levels were correlated with the course of disease for patients, fasting C-peptide, 2-hour postprandial C-peptide level, glycosylated hemoglobin level, and 24-hour urinary microalbumin excretion [39].

Li et al. conducted a study on a group of rats. The study explained the properties of exogenous BDNF in animals with streptozotocin-induced diabetic neuropathic pain. The research proved that continued intrathecal administration of BDNF to diabetic animals relieved mechanical and thermal hyperalgesia. What is more, administration of BDNF reduced the hyperexcitability of dorsal root ganglion neurons. This study suggests that BDNF may have a role in the treatment of painful diabetic neuropathy [40].

In summary, in the course of diabetes, the most important issue is the development of neurovascular complications. It was proved that a low serum level of BDNF was a risk factor for diabetic retinopathy. Moreover, in people with diabetic peripheral neuropathy, BDNF serum levels were lower in comparison with a group with diabetes without complications and a healthy control group [36,37,38]. There is also evidence for the therapeutic possibilities of BDNF. The injection of BDNF into the vitreous cavities of diabetic rats prevented dopaminergic amacrine cells from degeneration [38]. Moreover, the study showed that continued intrathecal administration of BDNF to diabetic rats relieved hyperalgesia [40]. 

The studies above suggest that a low level of BDNF may be a risk factor of diabetic neurovascular complications and that the administration of exogenous BDNF may have a role in the treatment of diabetes complications.

## 5. How Can We Increase BDNF Levels Behaviorally?

Due to the many beneficial aspects of BDNF, not only in relation to brain function but also its metabolic effects, a question then arises: how can the level of BDNF be increased? The experimental data showed above proved that BDNF can be administered exogenously. However, this method of supplementation is still experimental and not fully explored in humans. It seems that endogenous BDNF level may be increased through physical activity. Physical activity is a factor that has a positive influence not only on the control of body mass, insulin resistance and metabolic control but also on the CNS [41,42,43]. The relationship between physical activity and brain functions has been described in many publications. 

### 5.1. Experimental and Animal Studies 

Eslami et al. conducted a research study on male Wistar rats. Their study showed that diabetes reduced the expression of BDNF in the sensory and motor roots. In addition, this phenomenon can be reversed through endurance training. This study suggested that endurance training may have the potential to compensate for the reduced expression of BDNF in diabetes neuropathy [44]. Tang et al. researched the effects of ladder climbing and aerobic treadmill exercises on learning and memory ability in diabetic rats. They also studied the possible mechanisms of this phenomenon. The diabetic control group showed decreased expressions of BDNF and the cAMP-response element binding protein (CREB) gene in the hippocampus and decreased learning and memory ability compared with the normal control group. The groups of rats who performed exercises (ladder climbing, aerobic treadmill) showed upregulated expressions of BDNF and CREB in the hippocampus and increased learning ability. The diabetic loading ladder group showed upregulated expression of the tropomyosin receptor kinase B (TrkB) gene in the hippocampus, and their spatial memory ability was increased. The diabetic loading ladder group showed upregulated expressions of the TRKB and CREB genes in the hippocampus, compared to the diabetic aerobic treadmill group. Physical exercise has a positive influence on the learning ability of diabetic rats, and this phenomenon may be connected to the upregulation of the BDNF/TrkB/CREB signaling pathway [45].

### 5.2. Healthy Population 

A study on patients above 65 years old with dementia showed that patients who rode a bicycle for a minimum of 15 minutes daily, for 15 months, instead of taking part in recreational activities (e.g., playing cards, reading), as the control group did, achieved better results than the control group. They improved mainly in terms of memory and mobility [43].

Floel et al. conducted a study on a group of 75 healthy patients, 50–78 years old, which was intended to explore whether physical activities such as garden keeping, walking the stairs, etc., and also sport (measured with a physical activity questionnaire), parameters of aerobic exercises (maximum power measured on a cycle ergometer, speed of walk on a treadmill before reaching lactate threshold), or both factors were associated with better memory. This study also aimed to explain the mechanisms underlying the positive impact of physical activity on the memorizing process. The study considered age, sex, education, depression, alcohol consumption, and smoking. The results showed that the level of physical activity measured with the physical activity questionnaire (but not cardiovascular fitness) was associated with better memory. Physically active patients showed increased grey matter volume, especially in the prefrontal cortex and in the cingulate cortex, during MRI. This group also had in laboratory tests a higher concentration of a neurotrophin—Granulocyte-Colony Stimulating Factor (G-CSF). The neurotrophin G-CSF might be a factor responsible for memory improvement at least because of its possible influence on the increase in grey matter volume shown during MRI in the study. Physical activity level was not significantly correlated with BDNF level [46].

Babaei et al. conducted a study on well-trained individuals and people living a sedentary lifestyle, showing that the basic BDNF concentration in serum was significantly lower in the well-trained group than in the control group. Moreover, this study demonstrated an inverse correlation between BDNF concentration and maximal oxygen uptake (VO2max), and a positive correlation with participants’ BMI. In both groups, aerobic and anaerobic exercises increased the BDNF level to about its initial value. Well-trained people had better results in a picture recall memory test. It was demonstrated that long-term and habitual physical activity could cause lower peripheral BDNF concentration, but positively influence memory functions [11]. 

Cho et al. conducted studies on a group of 18 healthy college men from the Department of Physical Education. The students’ blood was examined before and after a test for maximal oxygen uptake (VO2max), considered as a measure of the aerobic efficiency of the organism. There was no correlation between VO2max and platelets number before and after the exercises. An increase in platelets number and BDNF concentration in serum, plasma and platelets immediately after the exercises was noticed. The study showed a negative correlation between VO2max and BDNF concentration in plasma, serum and platelets at rest, and a positive correlation between the abovementioned factors immediately after the exercises [47].

### 5.3. Diabetes

Tonoli et al. examined the effects of two different intensities of exercise (high-intensity and continuous medium-intensity) on neurotrophins in people with T1D. Serum BDNF was evaluated pre and post all the exercises and after recovery. BDNF levels increased significantly after both exercise intensities. What was interesting was that the BDNF increase had a dose-response effect for exercise intensity [48]. Physical activity may help in maintaining or improving neurological health in people with diabetes. Moreover, Brinkmann et al. in their research examined the level of neurotrophic factors before and after exercise in the blood of elderly people with type 2 diabetes. They proved that depending on the exercise mode, acute submaximal exercise can increase levels of neurotrophic factors (BDNF, VEGF) in the blood of elderly T2DM patients [49]. Miyamoto et al. came to similar conclusions to those of previous authors. They examined the impact of prolonged neuromuscular electrical stimulation on blood parameters in type 2 diabetes. They concluded that patients with type 2 diabetes mellitus during the eight-week neuromuscular electrical stimulation training program had greater changes in BDNF plasma levels than the control intervention [50]. However, Lee et al. came to a different conclusion than previous researchers. During their study, they proved that adolescents with type 2 diabetes mellitus and a control group had similar resting BDNF levels. After 12 weeks of aerobic exercise, when the experiment stopped, there was no significant increase in the resting BDNF levels in the group of patients with type 2 diabetes mellitus [29]. 

**Figure 2:**
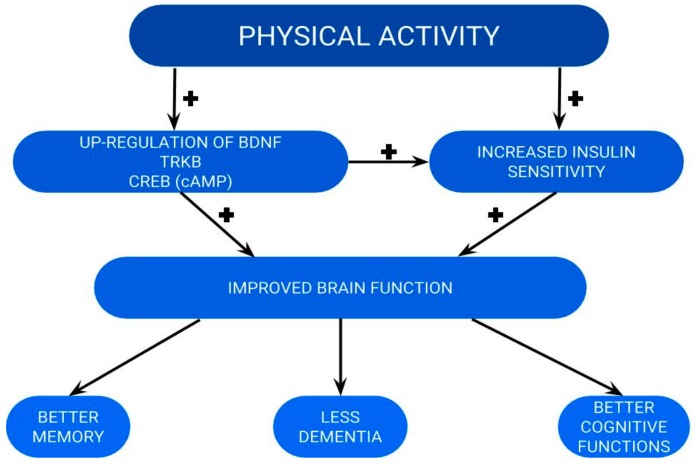
Physical activity and brain function—a place for BDNF action.

In humans, the levels of BDNF in the central nervous system are unknown because such research is technically difficult to perform. The levels of peripheral BDNF in humans are not synonymous with the level of BDNF in the central nervous system. Studies show that diabetes reduces the concentration of BDNF in the central nervous systems of animals, and this phenomenon can be reversed through endurance training. We may assume that the situation is similar in the human population. However, further studies are needed. A summary of this section is presented in Figure 2 and Supplementary Table S1.

## 6. Summary

Despite numerous publications being available in medical article databases, there is still not enough knowledge on the relationship between neurotrophins and physical activity in patients with diabetes. There are no unambiguous data on initial neurotrophin levels in patients with diabetes. We know that physical activity is a factor that improves cognitive functions such as memorization and that it improves mobility. Physical exercises contribute to increasing BDNF secretion. However, the basic BDNF concentration is lower in well-trained people. There is a hypothesis that the BDNF level increases to improve patients’ metabolism. BDNF may be a factor linked to a beneficial effect of physical activity on the neurological status of diabetic patients. Another known factor that influences the increase in BDNF secretion is dietary restrictions. BDNF may have an influence on improving fasting glucose and postprandial blood glucose, as well as on hyperinsulinemia reduction. BDNF may have a positive impact on the effect of insulin resistance.

## Supplementary Materials

The following are available online at https://www.mdpi.com/1422-0067/21/3/841/s1.

## Abbreviations

CNS	Central Nervous System
HBA1C	Glycated Hemoglobin
NGF	Nerve Growth Factor
BDNF	Brain-Derived Neurotrophic Factor
NT	Neurotrophin
HOMA-IR	Homeostatic Model Assessment for Insulin Resistance
BMI	Body Mass Index
CRP	C-reactive protein
WBC	White blood cells
CT	Computed Tomography
IR	Insulin Resistance
G-CSF	Granulocyte-Colony Stimulating Factor
VO2max	Test for Maximal Oxygen Uptake
p75NTR	p75NTR p75 NT receptor
TrkB	receptor tyrosine kinase B
CREB	cAMP-response element binding protein
