# Supplementary material for: Brain-Derived Neurotrophic Factor and Diabetes
Source: Int J Mol Sci. 2020 Jan 28;21(3):841. doi: 10.3390/ijms21030841 (PMC7037992; doi:10.3390/ijms21030841)
Supplement: Supplementary file 1 [file ijms-21-00841-s001.pdf]

Table 1. Methods for increasing BDNF levels in mammals and their beneficial effects.

**1. Exogenous injection of BDNF**				
**Research**	**Material**	**Methods**	**Effect on serum BDNF**	**Other effects**
Ono M et al [20]	mice	Subcutaneous administration of BDNF	No data	-decrease of elevated blood glucose level,
Mitsugu et al. [23]	obese diabetic mice	subcutaneous administration of BDNF to obese diabetic mice	No data	-decrease glucose concentration,<br>-decrease concentration of pancreatic glucagon<br>- increase concentration of pancreatic insulin,<br>-increase beta cell producing insulin in pancreatic islet, -decrease non-producing insulin cells area
Kuroda et al. [24]	obese rats	administration of exogenous BDNF	No data	-improve glucokinase activity,<br>-decrease hepatic gluconeogenesis,<br>-improve insulin sensitivity,<br>-decrease level of fasting glucose and postprandial blood glucose<br>-decrease hyperinsulinemia
Meek et al [25]	mice	intracerebroventricular or or into the ventromedial hypothalamic nucleus injections of either BDNF or its vehicle	No data	- decrease hyperglycemia, -decrease blood glucose levels -suppress hepatic glucose production through inhibition of glucagone
Masaaki Seki et al.[38]	streptozotocin-induced diabetic rats	intraocular administration of BDNF	No data	-prevention dopaminergic amacrine cells from degeneration<br>-potential role in the treatment of early retinal neuropathy
Lei li et al. [40]	diabetic rats	continued intrathecal administration of BDNF	No data	-alleviation of mechanical and thermal hyperalgesia<br>-reduce hyperexcitability of dorsal root ganglion neurons<br>-potential role in the treatment of painful diabetic neuropathy

**2. Dietetary restrictions**				
**Research**	**Material**	**Methods**	**Effect on BDNF**	**Other effects**
Duan et al. [22]	Mice	dietetary restrictions	increase BDNF production in brain cells	decrease concentration of glucose, insulin, and leptin in mice whose level of these factors was increased
**3. Physical activity**				
**Research**	**Material**	**Methods**	**Effect on BDNF**	**Other effects**
Babaei et al. [11]	well-trained individuals and people living a sedentary lifestyle	Comparison of well-trained individuals and people living a sedentary lifestyle aerobic and anaerobic exercises	-basic BDNF concentration in serum is significantly lower in the well-trained group than in the control group<br>-In both groups aerobic and anaerobic exercises increased BDNF level about its initial value	Well-trained people had better results in a picture recall memory test.
Lee et al. [29]	adolescents with type 2 diabetes mellitus	12 weeks aerobic exercise	-adolescents with type 2 diabetes mellitus and control group had similar resting BDNF levels.<br>-after the 12 weeks aerobic exercise,, there was no significant increase in the resting BDNF levels in the patients with type 2 diabetes mellitus group	
Tonoli et al. [31]	patients with type 1 diabetes	high-intensity exercise	-serum BDNF levels were significantly higher in people with type 1 diabetes compared with the control healthy group.<br>-in both groups BDNF increased after exercise.	
Cancela JM et al. [43]	patients with dementia	riding a bicycle for minimum 15 minutes instead of taking part in recreational activities		-improved memory<br>-improved mobility

Rasoul Eslami et al. [44]	rats	endurance training	-diabetes reduce the expression of BDNF in the sensory and motor roots.<br>-this phenomenon can be reversed through endurance training.	
Tang et al. [45]	rats	weight-bearing ladder and aerobic treadmill exercise	upregulate expression of BDNF and CREB in hippocampus	- learning ability increased
Floel et al. [46]	healthy patients	comparison of patients according to physical activity, measured with physical activity questionnaire, parameters of aerobic exercises, or both factors	-physically active patients had higher concentration of neurotrophin – a Granulocyte-Colony Stimulating Factor (G-CSF)<br>-physical activity level was not significantly correlated with the BDNF level	- Physically active patients showed increased grey matter volume
Hyun-chul Cho et al. [47]	healthy patients	treadmill VO2max performance	-increase in BDNF concentration in serum, plasma and platelets immediately after exercises	
Tonoli et al. [48]	patients with type 1 diabetes	two different intensities of exercises (high-intensity and continuous medium-intensity)	-BDNF levels increased significantly after both exercise intensities. The BDNF increase had a dose-response effect for exercise intensity	
Brinkmann et al. [49]	elderly people with type 2 diabetes	examine the level of neurotrophic factors before and after exercise	-depending on the exercise mode, acute submaximal exercise can increase levels of neurotrophic factors (BDNF, VEGF)	
